# Role of the Metal-Oxide Work Function on Photocurrent Generation in Hybrid Solar Cells

**DOI:** 10.1038/s41598-018-21721-2

**Published:** 2018-02-23

**Authors:** Chawloon Thu, Philipp Ehrenreich, Ka Kan Wong, Eugen Zimmermann, James Dorman, Wei Wang, Azhar Fakharuddin, Martin Putnik, Charalampos Drivas, Aimilios Koutsoubelitis, Maria Vasilopoulou, Leonidas C. Palilis, Stella Kennou, Julian Kalb, Thomas Pfadler, Lukas Schmidt-Mende

**Affiliations:** 1 Department of Physics, University of Konstanz, POB 680, 78457 Konstanz, Germany; 2 Cain Department of Chemical Engineering, 3307 Patrick Taylor Hall, Louisiana State University, Baton Rouge, LA 70803 USA; 3 Department of Chemical Engineering, University of Patras, Patras, 26504 Greece; 4 Department of Physics, University of Patras, 26504 Patras, Greece; 5 Institute of Nanoscience and Nanotechnology, National Center for Scientific Research, Demokritos, Agia Paraskevi, 15310 Athens, Greece

## Abstract

ZnO is a widely used metal-oxide semiconductor for photovoltaic application. In solar cell heterostructures they not only serve as a charge selective contact, but also act as electron acceptor. Although ZnO offers a suitable interface for exciton dissociation, charge separation efficiencies have stayed rather poor and conceptual differences to organic acceptors are rarely investigated. In this work, we employ Sn doping to ZnO nanowires in order to understand the role of defect and surface states in the charge separation process. Upon doping we are able to modify the metal-oxide work function and we show its direct correlation with the charge separation efficiency. For this purpose, we use the polymer poly(3-hexylthiophene) as donor and the squaraine dye SQ2 as interlayer. Interestingly, neither mobilities nor defects are prime performance limiting factor, but rather the density of available states around the conduction band is of crucial importance for hybrid interfaces. This work highlights crucial aspects to improve the charge generation process of metal-oxide based solar cells and reveals new strategies to improve the power conversion efficiency of hybrid solar cells.

## Introduction

The concept of hybrid metal-oxide polymer solar cells is driven by the motivation of combining advantages of organic and inorganic solar cells^1,2^. Besides high power conversion efficiencies due to an efficient charge generation process, it is essentially important to demonstrate devices with high ambient- and photostability made of low cost and nontoxic materials^3–6^. In the past, there have been numerous research efforts to improve the low performance of hybrid solar cells with respect to purely organic or inorganic devices^1,2,7^. Studies on the influence of crystal defects in the metal-oxides, different architectures, surface modifications, distinct recombination, and different organic/inorganic layers revealed performance limitations that are different to purely organic or inorganic devices^1,2,7–16^. Though none of these studies fully explained the rather low performance, it is generally invoked that the charge generation process at a polymer metal-oxide interface is not very efficient^17^. For this reason, it is fundamentally important to identify and address limiting factors to efficient charge separation and collection. Only recently, it has been shown that the charge generation process in hybrid solar cells is significantly enhanced if exciton separation happens at the metal-oxide-dye interface, while excitons from the polymer are resonantly transferred to the dye^18^. Additionally, trap and surface states on the metal-oxide surface are found to have a large impact on charge separation and recombination while the large binding energies of bound charge pairs at the organic/inorganic interface are a significant obstacle for exciton dissociation and charge separation^19–23^. Inorganic n-type metal-oxides such as ZnO, TiO_2_, and SnO_2_ are frequently used as electron transport layers and p-type conjugated polymers such as P3HT, PCPDTBT, and spiro-OMeTAD are used as hole transport layers^7,15,24–28^. Within this material combination it is important to note that typical hole mobilities in P3HT (10^−6^–10^−4^ cm^2^ V^−1^ s^−1^)^29,30^, PCPDTBT (10^−4^–10^−3^ cm^2^ V^−1^ s^−1^)^31^ and spiro-OMeTAD (10^−4^–10^−3^ cm^2^ V^−1^ s^−1^)^32^ are orders of magnitude lower than electron mobilities in bulk ZnO (200–300 cm^2^ V^−1^ s^−1^)^33–35^, ZnO nanorods (1–30 cm^2^ V^−1^ s^−1^)^36,37^, ZnO nanowires (20–220 cm^2^ V^−1^ s^−1^)^38^, single ZnO nanowires (1000 cm^2^ V^−1^ s^−1^)^39^, TiO_2_ (0.1–1 cm^2^ V^−1^ s^−1^)^33^, or SnO_2_ (100–250 cm^2^ V^−1^ s^−1^)^33,40^. This large discrepancy in mobilities raises the question on the influence of space charge formation and the impact on device performance.

The impact of doping on metal-oxide properties and resulting consequences on solar cell performance have been studied extensively in the past^41,42^. By usage of a broad range of different dopants like Mg^43^, Sr^44^, Ca^45^, N^9,46^, Sn^42,47^ and Cs^48^ power conversion efficiencies have increased significantly. In general, improved open circuit voltages have been explained by an uplift in the conduction band or a decreased conductivity, while larger photocurrents are justified by rougher surfaces that enhance the interfacial area or by a reduced electron concentration and recombination at surface traps^9^. Although interfacial characteristics have been extensively investigated, little is known about the role of the metal-oxide bulk properties and its contribution towards interfacial charge separation. Herein, we investigate the effect of the bulk properties on the charge dynamics in hybrid solar cells using pristine and doped ZnO-P3HT as a model system. All cells are fabricated with a self-assembled monolayer (SAM) of the squaraine dye SQ2 acting as an interlayer between polymer and metal-oxide in order to not only track changes in surface characteristics but also to reduce the role of surface states. Interestingly, we find the influence of space-charges to be rather low. Our experiments show that a low work function of the metal-oxide favours a large driving force for exciton separation while intrinsic charges in the metal-oxide reduce the photocurrent generation.

## Experimental Methods

**Figure 1:**
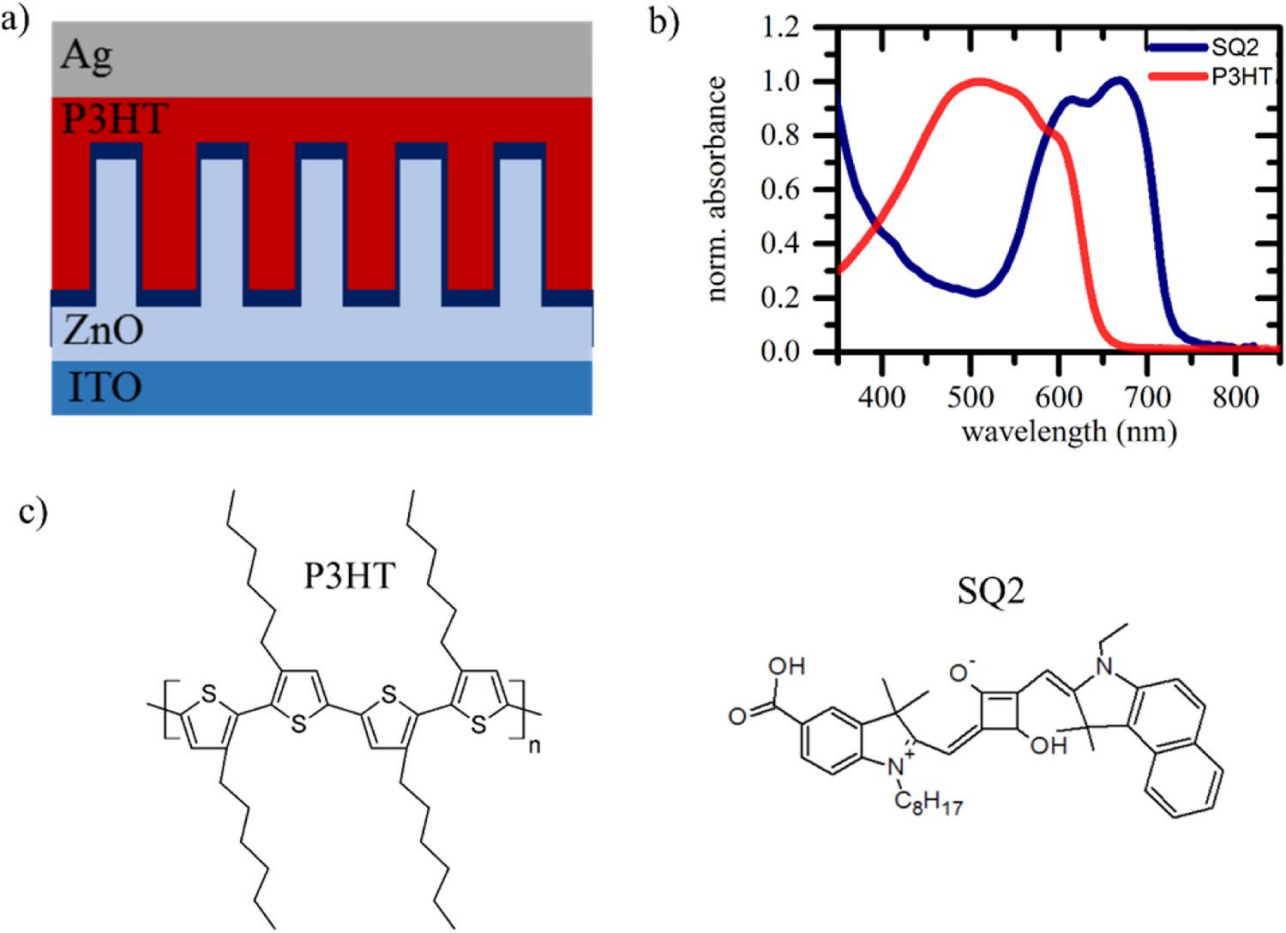
(**a**) Schematic drawing of the investigated hybrid solar cell employing ZnO nanowires with variant Sn doping concentrations. The ZnO surface is decorated with a SQ2 dye monolayer (indicated in dark blue). (**b**) Normalized absorbance spectra of the donor polymer P3HT and the dye molecules SQ2 acting as photoactive materials. Their chemical composition is shown in (**c**).

The hybrid solar cells fabricated in this work consist of an ITO/ZnO nanowire (NW)/SQ2/P3HT/Ag or ITO/Sn:ZnO NWs/SQ2/P3HT/Ag structure which is shown in Fig. 1. Firstly, ITO-coated glass substrates (Solaronix, 15 Ω/sq, size 14 × 14 mm²) were cleaned with acetone and isopropanol for 5 min in an ultrasonic bath. Subsequently, the samples were dried with nitrogen and subjected to oxygen plasma for 7 min. A 20 nm thick ZnO seed layer was deposited via a sol-gel spin coating route at room temperature using a zinc precursor solution containing 0.3 M zinc acetate dehydrate (Sigma Aldrich), and ethanolamine (Sigma Aldrich) in 2-methoxyethanol (Sigma Aldrich) which was spun at 2000 rpm for 40 s. The seed layers were annealed at 300 °C for 10 min. For XRD analysis, the samples were prepared on glass substrates. Pure ZnO and Sn doped ZnO nanowires are grown on top of this seed layer via a hydrothermal synthesis route. [31–33]. For pristine ZnO NWs a 25 mM ZnO solution with zinc nitrate hexahydrate (Sigma Aldrich) and methenamine (Merck KGaA) were prepared in in DI water. For doping, 1–3 mol % tin (ΙV) chloride hydrate (Alfa Aesar) was added to this solution. After the ZnO solution has been preheated at 95 °C for 90 min, as prepared ZnO films were immersed into the solution with the seed layer face down for 25 min at 95 °C. The nanowires were then washed with water and subsequently annealed at 250 °C for 30 min. Afterwards, the nanowire surface was modified with a SQ2 dye monolayer using a 0.2 mM dye bath in ethanol for 13 min. The unanchored dye was removed with an isopropanol wash. Before spin coating P3HT (Rieke Metals, 69 kDa − 30 mg/ml dissolved in chlorobenzene) at 1500 rpm for 110 s, the samples were pre-wetted with chlorobenzene. Subsequent post-annealing at 120 °C for 5 min was carried out in order to remove excess solvent. All processes were performed under ambient condition. Finally, 130 nm Ag was deposited on the films through a shadow mask by thermal evaporation under a vacuum pressure of <5 × 10^−6^ mbar.

### Characterization

The surface morphology and structural properties of nanowires were characterized using a scanning electron microscopy (SEM) (Zeiss Crossbeam 1540 SEM) at 6 keV accelerating voltage. Additionally, X-Ray and ultra-violet photoemission spectroscopy (XPS and UPS) measurements were carried out in order to characterize the chemical composition of their surfaces as well as the electronic structure of the valence band (VB), respectively. For this purpose, ZnO NWs on ITO substrates are used with nominal Sn^4+^ doping of 0 mol%, 1 mol%, 2 mol% and 3 mol%. All samples were measured without any further cleaning treatment prior to their introduction into an ultra-high vacuum chamber. For XPS, the Mg Kα line at 1253.6 eV (12 kV with 20 mA anode current, not monochromized) is used in combination with an analyser (Leybold EA-11) with a pass energy of 100 eV, giving a full width at half maximum (FWHM) of 1.3 eV for the Au 4f_7/2_ peak. The analysed area was approximately a 2 × 5 mm^2^ rectangle, positioned near the geometric centre of each sample. XPS analysis was carried out at 0° take-off angle (normal to the sample surface). In all XPS spectra, the binding energy (BE) of the predominant aliphatic contribution to the C 1 s peak at 284.8 eV was used as a measured BE reference. For the UPS measurements, the He I excitation line (21.2 eV) was used, and a negative bias of 12.23 V was applied to the specimen in order to separate the high binding energy cut-off from the analyser. The error in our UPS measurements is 0.1 eV. Since ZnO is a photosensitive material all UPS spectra were taken prior XPS measurements. Crystallographic properties were carried out by X-ray diffraction (XRD) via a Bruker AXS D8 Advance diffractometer and recorded at 2θ range 20–80° at a rate of 0.004 °/s with a Cu Kα source. A Keithley 2400 source meter was used for current density-voltage (J-V) and external quantum efficiency (EQE) measurements using a home-built LabVIEW program. The devices were illuminated with a LOT-Oriel LS0106 solar simulator equipped with an AM 1.5 G filter using a light intensity of 100 mWcm^−2^. For light intensity dependent J-V measurements a series of neutral density filters was used for attenuation of the incident light intensity. The cell area of 0.125 cm^2^ was defined through a shadow mask after calibrating with a Fraunhofer Institute certified Si reference solar cell using a KG5 filter. For EQE measurements the solar cells were illuminated with monochromatic light (LOT-Oriel Omni 150 monochromator in combination with a 150 W Xe lamp). The acquired absorption spectra were recorded using a Cary 5000 UV-VIS-NIR spectrometer equipped with an integrating sphere. For conductivity measurements a platinum-iridium (PtIr) (4:1) tip was used as top contact on Zn NW/ITO structures. In these measurements, samples are investigated in dry nitrogen and contacted to a Keithley 2401 serving as a voltage source and measurement unit for transient currents.

## Results

Figure 1(a) depicts the device structure of our hybrid nanostructured solar cell architecture (ITO/ZnO NWs/SQ2/P3HT/Ag). The NWs are 200 nm in length with a diameter of 20–30 nm independent of doping concentration (see Figure S1 in the Supplementary Information). This similarity in morphology, microstructure composition and shape make them an ideal model system for the investigation on the role of dopants in metal-oxide based hybrid solar cells. Figure 1(b) shows the absorption spectra of employed organic absorber materials, namely SQ2 and P3HT whose chemical structures are drawn in Fig. 1(c).

**Figure 2:**
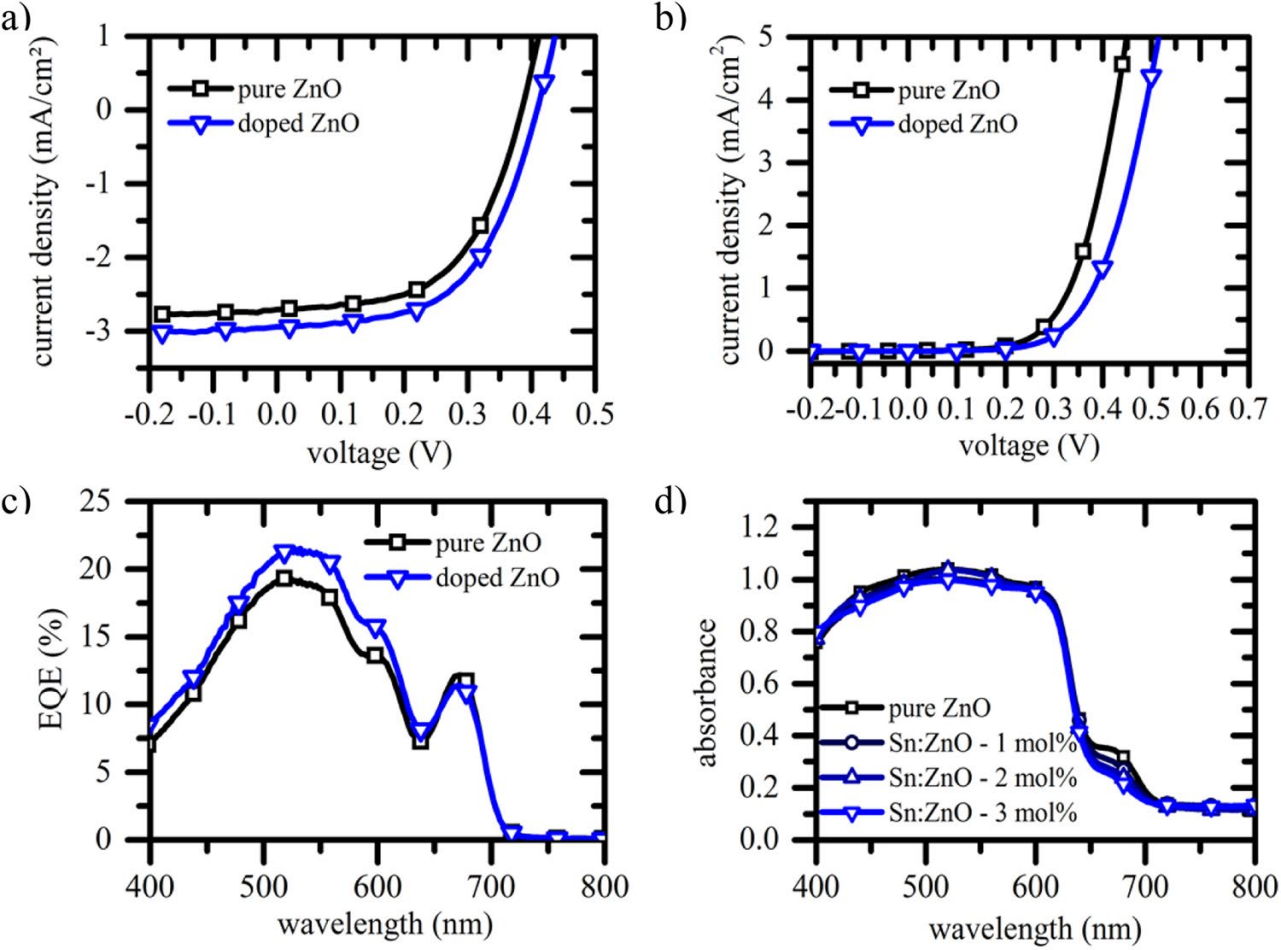
(**a**) J−V characteristics of hybrid solar cell under illumination using ZnO nanowire arrays with 3 mol% Sn^4+^ doping (blue curve) and without doping (black curve) (AM 1.5G, illumination 100 mWcm-2); (**b**) J-V characteristics of the full device in dark. (**c**) EQE spectra of hybrid nanostructured devices; (**d**) absorption spectrum of the full structure for the pure and doped ZnO nanowire systems after decoration with SQ2-P3HT and evaporation of Ag.

Table 1 summarizes the solar cell characteristics of devices made of pristine and doped ZnO. We present an average performance and champion cells (brackets) out of more than 40 cells from 12 independent batches for each kind^49^. Upon Sn doping, we see a strong rise in *J*_*SC*_ which results in an improvement of power conversion efficiency by more than 30%. In Fig. 2, representative J-V curves are shown for non-doped and doped (3 mol% Sn) ZnO based cells under illumination (a) and under dark conditions (b). In Fig. 2(b) we observe an increase in the built-in potential upon Sn doping, while the series resistance is enhanced. This is a phenomenon typically not observed under illumination and indicates its charge carrier density dependence. Furthermore, external quantum efficiency (EQE) measurements (Fig. 2(c)) reveal an enhanced photocurrent due to improved contributions over a broad spectral range (425–600 nm), a range that is attributed to P3HT. In contrast, for longer wavelengths where solely SQ2 is absorbing, photocurrent contributions are almost indistinguishable or slightly reduced for Sn doped NWs. In this context, absorbance measurements on the full device structure (Fig. 2d) show a notable reduction of dye absorption for doped devices. A lower dye coverage can result from a changed surface stoichiometry of the metal-oxide and could explain reduced photocurrent contributions of SQ2. Hence, the improved photocurrent contribution by the polymer is even more remarkable since exciton transfer to the dye is seen to be superior compared to direct exciton splitting at a polymer metal-oxide interface^18^. We further track changes in surface properties by means of XPS and compare results to XRD and high resolution TEM (HRTEM) data. As shown in the Supplementary Information, XRD measurements reveal a loss in crystallinity upon Sn doping while XPS spectra are very similar and indicate only traces of SnO_x_ on the surface with otherwise similar elemental composition. Note that Sn is not clearly visible on the XPS wide scan due to its very low concentration and distribution in the NWs. Indium of the substrate is not present either, which implies that the nanowires are closely packed, as it is confirmed by the electron microscopy observations.

**Table 1:** Solar cell characteristics presented here are the statistical average of more than 40 solar cells of each kind out of 12 different and independent batches. Champion cell parameters are written in brackets.

Sn doping	*V*_*oc*_ (V)	*J*_*sc*_ (mA/cm^2^)	FF (%)	η (%)
0%	0.34 (0.44)	2.29 (2.64)	51 (55)	0.41 (0.64)
1%	0.37 (0.41)	2.71 (3.10)	54 (57)	0.57 (0.72)
2%	0.36 (0.39)	2.89 (3.26)	53 (56)	0.56 (0.70)
3%	0.38 (0.41)	2.77 (3.14)	52 (57)	0.53 (0.72)

**Figure 3:**
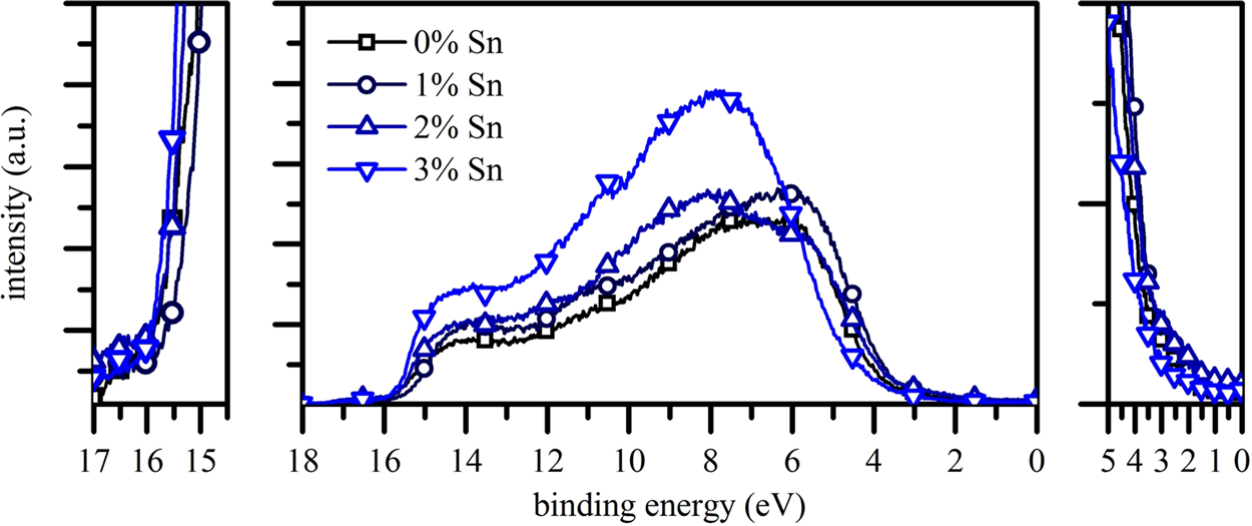
Middle: Full UPS spectra for all samples; Left: zoom-in of high binding energy cut-off in order to determine the sample work function (solid line); Right: zoom-in of low binding energy cut-off.

To investigate the electronic structure (valence band and work function) of the pristine and doped ZnO, UPS measurements were performed (Fig. 3). The middle part shows full spectra of the ZnO NWs while plots on the left and right are magnifications of the cut-off edges. The high binding energy cut-off depicts the work function (WF) by an intersection of a tangent line with the binding energy baseline. The WF of pristine NWs is found to be 5.3 ± 0.1 eV, which is significantly larger compared to a ZnO film (4.1 ± 0.1 eV – see Supplementary Information). This increase is probably due to the crystallinity of the NWs and the high concentration of O on the NW surface. A small increase in the WF of approximately 100–200 meV (in the order of the experimental error) was observed upon doping. In contrast, the low binding energy cut-off represents the valence band maximum (VBM) onset with respect to the Fermi level. For pure ZnO NWs, we extract a VBM onset binding energy of 3.3 ± 0.1 eV. No significant dependence of the VBM is observed, as expected for these doping levels. Only a small shift (i.e. increase of the VBM) of ~200 meV is noted up to the 3% doping.

The analysis of the full UPS spectrum reveals some additional interesting observations on the ZnO composition. In general, ZnO exhibits two characteristic peaks around 6 eV and 11 eV. The former can be attributed to 2p orbitals of oxygen from Zn-O bonds while the latter results from Zn 3d orbitals. In our NWs, both peaks are not very pronounced. In addition, the spectral shape shifts continuously towards higher binding energies upon doping and a broad peak at 8 eV becomes dominant. This signal has been correlated with a hybridization of Zn 4s-O 2p orbitals^50,51^ and gains in intensity and contribution to the overall spectra as Sn doping increases. This enhancement of hybridization between the cation’s *s*-orbitals and the *p*-orbitals of neighbouring oxygen atoms in the Sn doped NW spectra is expected to contribute in overcoming the electron localization bottleneck and facilitating electron transfer. We suggest that Sn doping may favour orbital hybridization as a result of charge transfer between Sn and ZnO. Note that for the NW spectra, an upward binding energy shift is observed compared to thin film spectra, which is attributed to surface band bending, as well as an overall spectral broadening and an increase of the density of states of the hybridized metal and oxygen orbitals.

As our XRD results suggest the growth of the NWs is always in (0002) direction and is independent of doping concentration, we can conclude that crystal growth is still largely preserved upon doping. However, a strong hybridization of Zn 4 s - O 2p orbitals points to a broad density of energetically lower lying states. This has been proposed and demonstrated experimentally for a variety of metal-oxide based materials^52,53^.

**Figure 4:**
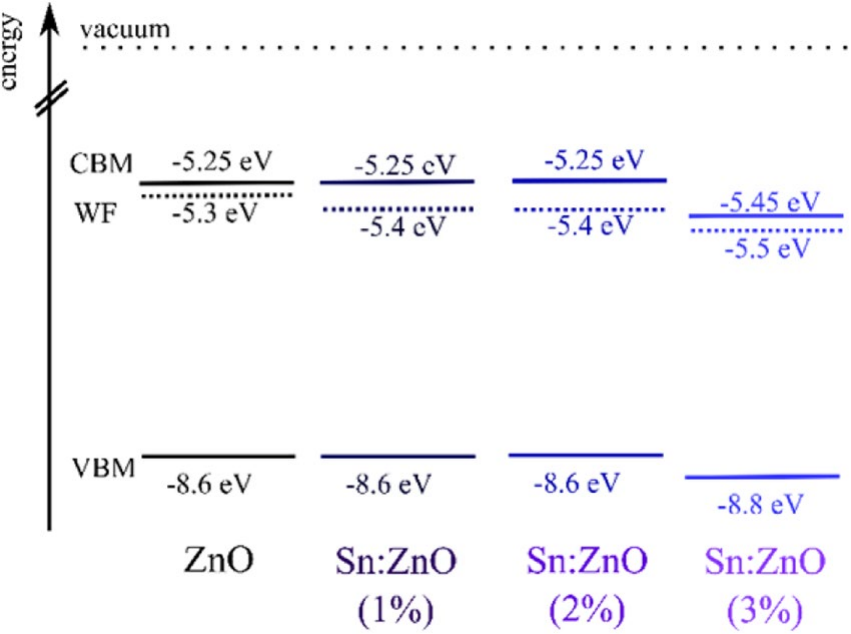
Energetic landscape in ZnO-NWs with doping concentrations of 0–3 mol% in the growth solution. Work function (WF) and valence band maxima (VBM) are obtained from UPS and conduction band minima (CBM) by substracting the absorption onset (energy band gap) from the VBM.

To further determine the energetic landscape in ZnO we measured the conduction band minimum (CBM) by means of absorbance measurements (Figure S6, Supplementary Information). The absorption onset is only marginally changed upon doping and the bandgap is around 3.35 eV for all doping concentrations in this study. In combination with the UPS data, the CB is located only ~50–150 meV above the Fermi level suggesting an excellent interfacial alignment for facile electron transport and predominant n-type behavior of the doped and undoped ZnO NWs.We plot the overall energetic landscape from absorbance and UPS measurements in Fig. 4. Although one would expect a small but systematic change in the V_OC_ as the CBM and the WF change upon doping, we do not observe such a significant dependence (Table 1). We note that the WF is relatively large for the crystalline NWs, though it can be explained by its dependence on different crystallographic directions^54^. Another possible explanation is a higher concentration of oxygen generally observed at the surface of the NWs, which is known to cause an increase in WF^55,56^. In our experiments, however, it is not possible to quantify individual contributions of different crystal faces to the measured WF.

**Figure 5:**
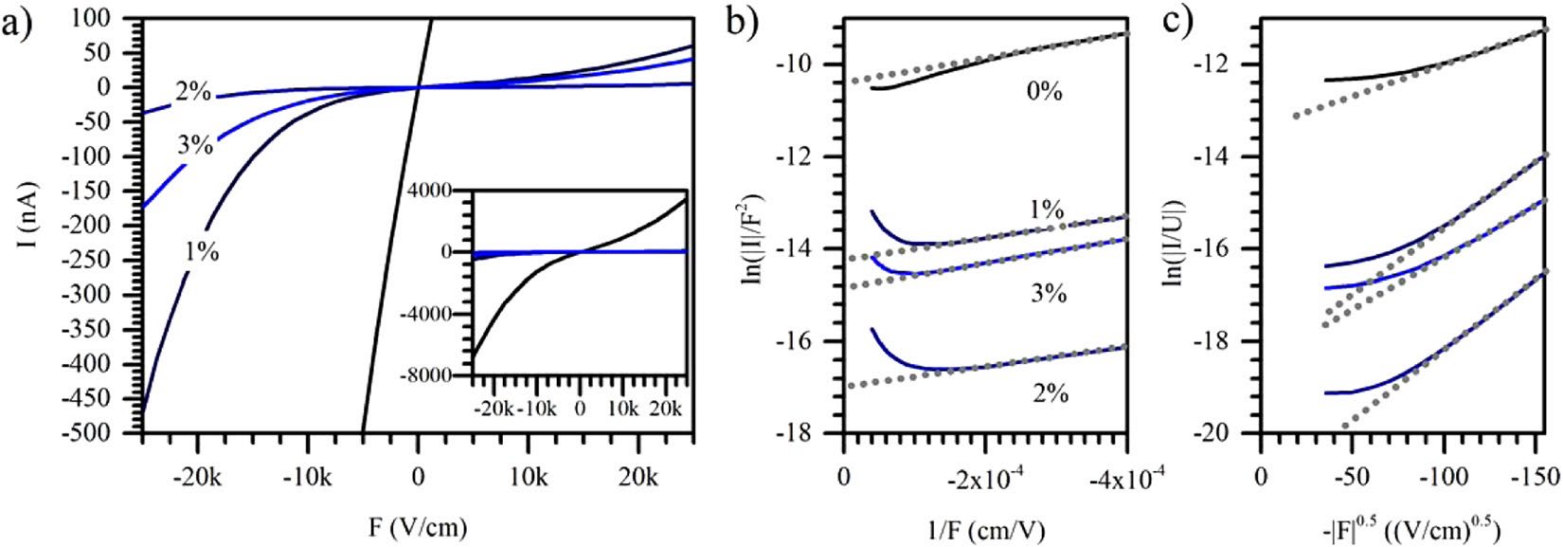
IV-characteristics of PtIr/ZnO/ITO layer diode; (**a**) linear plots; inset: linear plot for all 4 samples on larger scale on the current axis; (**b**) Fowler-Nordheim plot; (**c**) Poole-Frenkel plot. In order to directly keep related to the investigated solar cell devices (**b** and **c**) show the negative field only where the electrons move from the ZnO-NWs to the ITO.

Sn doping of ZnO is expected to increase the conductivity since Sn^4+^ ions substitute Zn^2+^ ions in the crystal by providing two loosely bound electrons per dopant. In a low doping concentration limit, this results in an increased carrier mobility and ionisation of the Sn atoms on the Zn sites^57^. In contrast, higher doping concentrations may lead to lattice distortion, despite the small difference in ionic radii between Zn and Sn and more neutral defects in the crystal. Consequently, the probability of carrier scattering on such charged impurity centres or generated defects is increased in the lattice and the mobility is lowered^57,58^. Conductivity measurements on our pristine ZnO structures reveal a reduced conductivity upon Sn doping (Fig. 5) suggesting the formation of such neutral defects and scattering centres at grain boundaries. For a relatively low doping concentration (1–2 mol%), it drops about 2 orders of magnitude, whereas for higher dopant concentrations the conductivity is slightly enhanced again.

The contact resistances were taken from the linear plot and are calculated as R(0%) ≈ 250 kΩ, R(1%) ≈ 17 MΩ, R(2%) ≈ 250 MΩ, and R(3%) ≈ 25 MΩ, respectively. Between 0 and 0.2 V, a linear regime in the Fowler-Nordheim plot (Fig. 5b) indicates a thin insulating shell around all samples. This affects the transient current through the doped NWs, in particular at low fields^59,60^. Such thin shells cannot be detected by XRD and might be even amorphous. The identical slope in each curve indicates the presence of a barrier with similar properties (i.e. width and height) for all samples.

The Poole-Frenkel (Fig. 5c) behaviour is simplified as $\mathrm{ln}(\frac{I}{F})=\tau {N}_{c}(A\sqrt{F}-B)$, where τ is the momentum scattering time, N_c_ is the density of states in the conduction band, and A and B are constants containing parameters such as the effective mass, the static dielectric constant, and the energy level distribution of the traps^61^. The slope as well as the vertical axis intersection point are increased for 1 and 2 mol% doped samples and thus, the factor τN_c_ is enhanced. For a 3 mol% doping concentration this factor is decreasing again. In general, a higher doping concentration should result in a larger number of mobile electrons in the conduction band (increased N_c_) such that an increased slope points to an enhanced scattering time τ.

Upon such variation in the metal-oxide conductivity, improved solar cell performances could also be explained by a reduced influence of space-charge limitations. However, (as shown in the Supplementary Information) mobilities play only a minor role in performance enhancements since the space charge limited current region is unaffected and we observe a very efficient charge collection at short circuit condition for all devices while the FF is constant.

## Conclusion

In summary, we have investigated ZnO NW-P3HT hybrid solar cells as a model system where defects and surface properties of ZnO are varied via Sn-doping. Upon doping the WF increases while the photocurrent in solar cell devices is enhanced no matter which doping concentration is applied. Although dye absorption is reduced, photocurrent contributions from the polymer are improved. Based on previous work^18^, showing that energy transfer is favoured compared to direct electron injection, this is a surprising result. It indicates the crucial role of a large density of states close to the conduction band while a large number of free electrons in the metal-oxide prevent excitons in the polymer from successful and efficient dissociation. As a result, photocurrent contributions by the polymer are reduced.

Furthermore, neither mobilities nor defect states close to the conduction band are primary performance limiting factors in hybrid solar cells. Instead, the energetic position of the WF is decisive whether electron injection is possible or hindered.

Within this work we can show, that doping of the metal-oxide offers a promising strategy to tune the relative energetic position of the WF and therefore improve the charge separation efficiency for efficient hybrid solar cells. Our results reveal the potential of further device improvements if a deeper understanding on the role of metal-oxide properties is gained especially if the WF can be tuned without the need of dopants or reduction in crystallinity.

## Electronic supplementary material

Supplementary Information

## References

[1] Liu R (2014). Hybrid Organic/Inorganic Nanocomposites for Photovoltaic Cells. Materials.

[2] Wright M, Uddin A (2012). Organic—inorganic hybrid solar cells: A comparative review. Sol Energ Mater Sol C.

[3] Sun H, Weickert J, Hesse HC, Schmidt-Mende L (2011). UV light protection through TiO2 blocking layers for inverted organic solar cells. Sol Energ Mater Sol C.

[4] Hintz H (2011). Photodegradation of P3HT−A Systematic Study of Environmental Factors. Chem Mater.

[5] Snaith HJ, Schmidt-Mende L (2007). Advances in Liquid-Electrolyte and Solid-State Dye-Sensitized Solar Cells. Adv Mater.

[6] Grancini G (2012). Boosting Infrared Light Harvesting by Molecular Functionalization of Metal Oxide/Polymer Interfaces in Efficient Hybrid Solar Cells. Adv Funct Mater.

[7] Dorman JA (2014). Control of Recombination Pathways in TiO2 Nanowire Hybrid Solar Cells Using Sn4+ Dopants. J Phys Chem C.

[8] Green MA, Emery K, Hishikawa Y, Warta W, Dunlop ED (2015). Solar cell efficiency tables (version 46). Prog Photovoltaics: Research and Applications.

[9] Musselman KP (2014). Improved Exciton Dissociation at Semiconducting Polymer:ZnO Donor:Acceptor Interfaces via Nitrogen Doping of ZnO. Adv Func Mater.

[10] Liao W-P, Hsu S-C, Lin W-H, Wu J-J (2012). Hierarchical TiO2 Nanostructured Array/P3HT Hybrid Solar Cells with Interfacial Modification. J Phys Chem C.

[11] Beek WJE, Wienk MM, Janssen RAJ (2004). Efficient Hybrid Solar Cells from Zinc Oxide Nanoparticles and a Conjugated Polymer. Adv Mater.

[12] Greene LE, Law M, Yuhas BD, Yang P (2007). ZnO−TiO2 Core−Shell Nanorod/P3HT Solar Cells. J Phys Chem C.

[13] Rattanavoravipa T, Sagawa T, Yoshikawa S (2008). Photovoltaic performance of hybrid solar cell with TiO2 nanotubes arrays fabricated through liquid deposition using ZnO template. Solar Energy Materials and Solar Cells.

[14] Wang L, Liu Y, Jiang X, Qin D, Cao Y (2007). Enhancement of Photovoltaic Characteristics Using a Suitable Solvent in Hybrid Polymer/Multiarmed CdS Nanorods Solar Cells. J Phys Chem C.

[15] Wisnet A (2015). Defeating Loss Mechanisms in 1D TiO2-Based Hybrid Solar Cells. Adv Func Mater.

[16] Ren S (2011). Inorganic–Organic Hybrid Solar Cell: Bridging Quantum Dots to Conjugated Polymer Nanowires. Nano Lett.

[17] Weickert J, Schmidt-Mende L (2013). Perspective: Hybrid solar cells: How to get the polymer to cooperate?. APL Mater.

[18] Ehrenreich P (2015). Role of charge separation mechanism and local disorder at hybrid solar cell interfaces. Phys Rev B.

[19] Sevinchan Y (2016). Improving Charge Separation across a Hybrid Oxide/Polymer Interface by Cs Doping of the Metal Oxide. Adv Mater Interfaces.

[20] Wu G, Li Z, Zhang X, Lu G (2014). Charge Separation and Exciton Dynamics at Polymer/ZnO Interface from First-Principles Simulations. J Phys Chem Lett.

[21] Lim K-G (2014). Boosting the Power Conversion Efficiency of Perovskite Solar Cells Using Self-Organized Polymeric Hole Extraction Layers with High Work Function. Adv Mater.

[22] Lim K-G (2016). Self-Doped Conducting Polymer as a Hole-Extraction Layer in Organic–Inorganic Hybrid Perovskite Solar Cells. Adv Mater Interfaces.

[23] Haque MA, Sheikh AD, Guan X, Wu T (2017). Metal Oxides as Efficient Charge Transporters in Perovskite Solar Cells. Adv Energ Mater.

[24] Krumm M, Pawlitzek F, Weickert J, Schmidt-Mende L, Polarz S (2012). Temperature-stable and optically transparent thin-film zinc oxide aerogel electrodes as model systems for 3D interpenetrating organic-inorganic heterojunction solar cells. ACS Appl Mater Interfaces.

[25] Eck M (2014). Improved efficiency of bulk heterojunction hybrid solar cells by utilizing CdSe quantum dot-graphene nanocomposites. Physical chemistry chemical physics: PCCP.

[26] Jotterand SA, Jobin M (2012). Characterization of P3HT:PCBM:CdSe Hybrid Solar Cells. Energy Proced.

[27] Lim K-G, Park SM, Woo HY, Lee T-W (2015). Elucidating the Role of Conjugated Polyelectrolyte Interlayers for High-Efficiency Organic Photovoltaics. ChemSusChem.

[28] Kim H, Lim K-G, Lee T-W (2016). Planar heterojunction organometal halide perovskite solar cells: roles of interfacial layers. Energ Environ Sci.

[29] Coakley KM (2005). Enhanced Hole Mobility in Regioregular Polythiophene Infiltrated in Straight Nanopores. Adv Func Mater.

[30] Goh C, Kline RJ, McGehee MD, Kadnikova EN, Fréchet JMJ (2005). Molecular-weight-dependent mobilities in regioregular poly(3-hexyl-thiophene) diodes. Appl Phys Lett.

[31] Albrecht S (2012). Fluorinated Copolymer PCPDTBT with Enhanced Open-Circuit Voltage and Reduced Recombination for Highly Efficient Polymer Solar Cells. J Am Chem Soc.

[32] Snaith HJ, Grätzel M (2006). Enhanced charge mobility in a molecular hole transporter via addition of redox inactive ionic dopant: Implication to dye-sensitized solar cells. Appl Phys Lett.

[33] Tiwana P, Docampo P, Johnston MB, Snaith HJ, Herz LM (2011). Electron Mobility and Injection Dynamics in Mesoporous ZnO, SnO2, and TiO2 Films Used in Dye-Sensitized Solar Cells. ACS Nano.

[34] Parmar NS, Corolewski CD, McCluskey MD, Lynn KG (2015). Potassium acceptor doping of ZnO crystals. AIP Advances.

[35] Noriega R (2010). Probing the electrical properties of highly-doped Al:ZnO nanowire ensembles. J Appl Phys.

[36] Law M, Greene LE, Johnson JC, Saykally R, Yang P (2005). Nanowire dye-sensitized solar cells. Nat Mater.

[37] Liao ZM (2011). Improved performance of ZnO nanowire field-effect transistors via focused ion beam treatment. Nanotechnology.

[38] Liu H, Peng R, Chu S, Chu S (2014). High mobility ZnO nanowires for terahertz detection applications. Appl Phys Lett.

[39] Zhang Q, Dandeneau CS, Zhou X, Cao G (2009). ZnO Nanostructures for Dye-Sensitized Solar Cells. Adv Mater.

[40] Thapa A (2014). TiO2 coated urchin-like SnO2 microspheres for efficient dye-sensitized solar cells. Nano Res.

[41] Fakharuddin A, Schmidt-Mende L, Garcia-Belmonte G, Jose R, Mora-Sero I (2017). Interfaces in Perovskite Solar Cells. Adv Energ Mater.

[42] Hoye RLZ, Musselman KP, MacManus-Driscoll JL (2013). Research Update: Doping ZnO and TiO2 for solar cells. APL Mater.

[43] Olson DC (2007). Band-Offset Engineering for Enhanced Open-Circuit Voltage in Polymer–Oxide Hybrid Solar Cells. Adv Funct Mater.

[44] Pachoumi O (2014). Improved Performance of ZnO/Polymer Hybrid Photovoltaic Devices by Combining Metal Oxide Doping and Interfacial Modification. J Phys Chem C.

[45] Wang M, Sun J-P, Suei S, Hill IG (2012). Optimizing the photovoltage of polymer/zinc oxide hybrid solar cells by calcium doping. J Appl Phys.

[46] Ehrler B (2013). Preventing Interfacial Recombination in Colloidal Quantum Dot Solar Cells by Doping the Metal Oxide. ACS Nano.

[47] Ye N (2010). Improvement of the performance of dye-sensitized solar cells using Sn-doped ZnO nanoparticles. J Power Sources.

[48] Sevinchan Y (2016). Charge Separation: Improving Charge Separation across a Hybrid Oxide/Polymer Interface by Cs Doping of the Metal Oxide. Adv Mater Interfaces.

[49] Zimmermann E (2014). Erroneous efficiency reports harm organic solar cell research. Nat Photon.

[50] Özgür Ü (2005). A comprehensive review of ZnO materials and devices. J Appl Phys.

[51] Ivanov I, Pollmann J (1981). Electronic structure of ideal and relaxed surfaces of ZnO: A prototype ionic wurtzite semiconductor and its surface properties. Phys Rev B.

[52] Medvedeva JE, Hettiarachchi CL (2010). Tuning the properties of complex transparent conducting oxides: Role of crystal symmetry, chemical composition, and carrier generation. Phys Rev B.

[53] Suntivich J (2014). Estimating Hybridization of Transition Metal and Oxygen States in Perovskites from O K-edge X-ray Absorption Spectroscopy. J Phys Chem C.

[54] Ozawa K (2016). Electron-Donor Dye Molecule on ZnO(101̄0), (0001), and (0001̄) Studied by Photoelectron Spectroscopy and X-ray Absorption Spectroscopy. J Phys Chem C.

[55] Hewlett RM, McLachlan MA (2016). Surface Structure Modification of ZnO and the Impact on Electronic Properties. Adv Mater.

[56] Heinhold R, Williams GT, Cooil SP, Evans DA, Allen MW (2013). Influence of polarity and hydroxyl termination on the band bending at ZnO surfaces. Phys Rev B.

[57] Dhamodharan P, Manoharan C, Dhanapandian S, Bououdina M, Ramalingam S (2015). Preparation and characterization of spray deposited Sn-doped ZnO thin films onto ITO subtracts as photoanode in dye sensitized solar cell. J Mater Sci-Mater El.

[58] Acharya AD (2012). Growth and characterization of nano-structured Sn doped ZnO. J Mol Struct.

[59] Zhang H, Solanki R, Roberds B, Bai G, Banerjee I (2000). High permittivity thin film nanolaminates. J Appl Phys.

[60] Lenzlinger M, Snow EH (1969). Fowler‐Nordheim Tunneling into Thermally Grown SiO2. J Appl Phys.

[61] Chiu, F.-C. A review on conduction mechanisms in dielectric films. *Adv Mater Sci Eng*, **2014** (2014).

