# Supplementary material for: Role of the Metal-Oxide Work Function on Photocurrent Generation in Hybrid Solar Cells
Source: Sci Rep. 2018 Feb 23;8:3559. doi: 10.1038/s41598-018-21721-2 (PMC5824951; doi:10.1038/s41598-018-21721-2)
Supplement: Supplementary file 1 — Supplementary Information [file 41598_2018_21721_MOESM1_ESM.pdf]

SUPPORTING INFORMATION

Role of the metal-oxide work function on photocurrent generation in metal-oxide polyer solar cells

Chawloon Thu,[1] Philipp Ehrenreich,[1,*] Ka Kan Wong,[1] Eugen Zimmermann,[1] James Dorman,[2] Wei Wang,[1] Azhar Fakharuddin,[1] Martin Putnik,[1] Charalampos Drivas,[3] Aimilios Koutsoubelitis,[4] Maria Vasilopoulou,[5] Leonidas C. Palilis,[4] Stella Kennou,[3] Julian Kalb,[1] Thomas Pfadler,[1] and Lukas Schmidt-Mende[1,*]

[1] Department of Physics, Department of Physics, POB 680, 78457 Konstanz, Germany.
[2] Cain Department of Chemical Engineering, 110 South Stadium Rd., Louisiana State University, Baton Rouge, LA, 70803 USA
[3] Department of Chemical Engineering, University of Patras, Patras, 26504 Greece
[4] Department of Physics, University of Patras, 26504 Patras, Greece
[5] Institute of Nanoscience and Nanotechnology, National Center for Scientific Research, Demokritos, Agia Paraskevi, 15310 Athens, Greece

Electron Microscopy & XRD

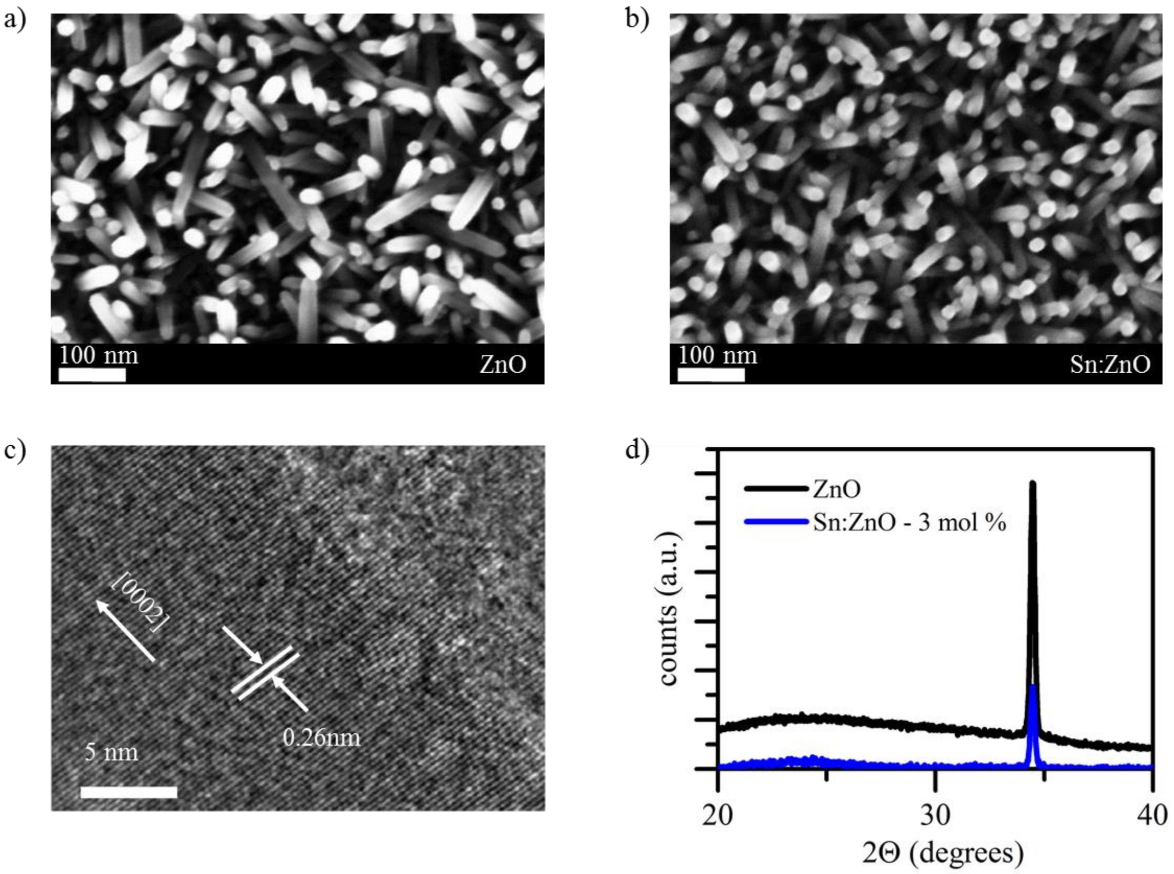

Figure S1| a) SEM image of ZnO NWs b) SEM image of Sn:ZnO NWs with 3 mol% doping c) Transmission electron microscopy (TEM) image of ZnO NWs. High resolution TEM (HRTEM) images reveal a d-spacing of 0.26 nm corresponding to the (002) plane of the wurtzite structure of ZnO. The growth axis of the nanowires corresponds to the c-axis of the wurtzite unit cell; d) X-ray diffraction patterns of undoped and doped ZnO NWs. Curves are shifted with respect to each other for clarity.

By incorporating Sn we do not find any apparent macroscopic structural changes in nanowires as seen in SEM images. Furthermore, high-resolution transmission electron microscopy (HRTEM) images on ZnO NWs show the interplanar spacing of 0.26 nm, which corresponds to the (002) plane of the wurtzite structure of ZnO. Also XRD data reveals that nanowires were preferentially oriented in (002) direction with its c-axis perpendicular to the substrate and indexed to the hexagonal Wurtzite structured of ZnO according to the JCPDS card No. 36-1451. Moreover, there is no peak shift observable and SnO or SnO_2 peaks are absent. It is evident that Sn^{4+} substituted successfully in native Zn^{2+} site without any aggregate formation. In contrast, we see that the intensity of the (002) plane peak is reduced which we attributed to a lower crystallinity. This can be done in this comparison, since geometry and morphology differences are minimal.

XPS:

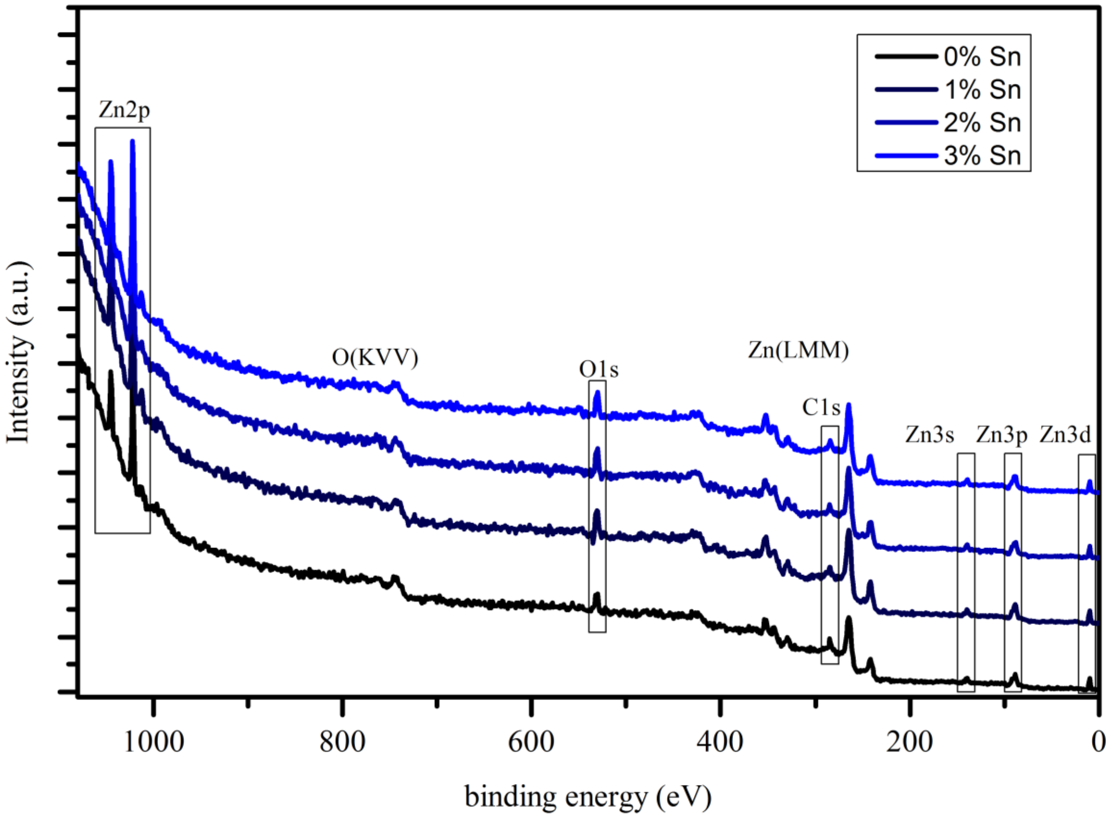

Figure S2| XPS wide scan for all samples

In Figure S2, XPS Wide Scans are shown. For all samples, we observe signals originating from C, O, and Zn. The presence of C we attribute to precursor residuals and atmospheric contamination, due to the exposure of the samples to the atmosphere before their introduction to the UHV chamber for the characterization. Indium from the ITO substrate, is not present, which implies that the nanowires are closely packed, as it is confirmed by electron microscopy observations. The Zn 2p peak appears as a doublet due spin orbit splitting. The Zn $2p_{3/2}$ peak is at a binding energy of 1021.7±0.1 eV for all the samples, which is attributed

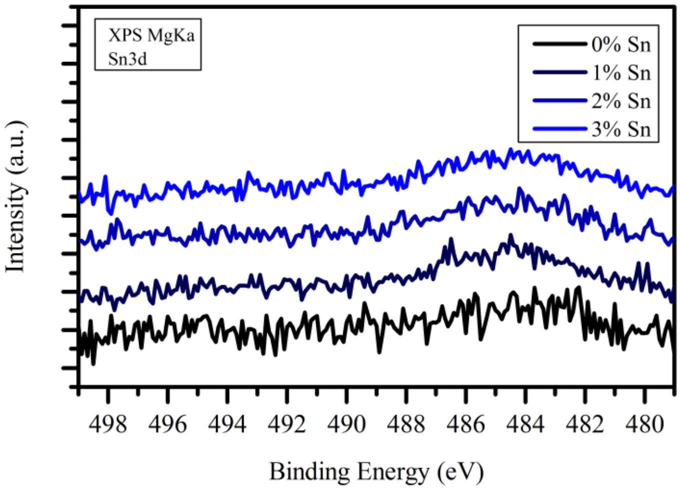

Figure S3| | XPS of Sn 3d peak for all the samples

to ZnO. No change is observable in all the samples which implies no chemical interaction with Sn.

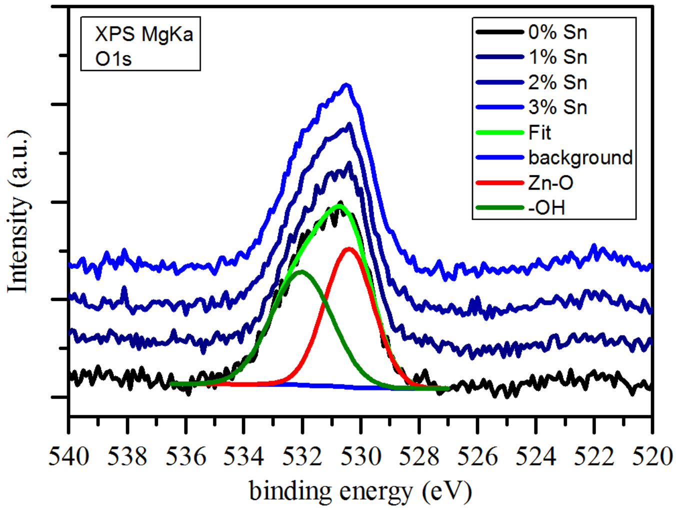

Figure S4| | XPS of the O1s peak for all samples

In Figure S3, the Sn 3d peak is presented. At the pristine sample a wide peak can be observed, which is attributed to the presence of Auger peaks of Zn. With increasing dopant concentration, only a small broadening of that peak is observable with a slight shift towards higher binding energies that could possibly be attributed to SnO_x though the low intensity indicates that only traces are present. Figure S4 presents the O 1s peak. The peak is asymmetric and large which we attribute to a superposition of two peaks, the first one with a binding energy of 530.4±0.1 eV, which is attributed to the Zn-O bond and the second one at about 532.0±0.1 eV, which is due to the OH (hydroxyls) from the atmosphere. No significant change in the overall shape and the two-peak component ratio is observed at the O1 s peak with Sn doping.

Absorbance spectroscopy

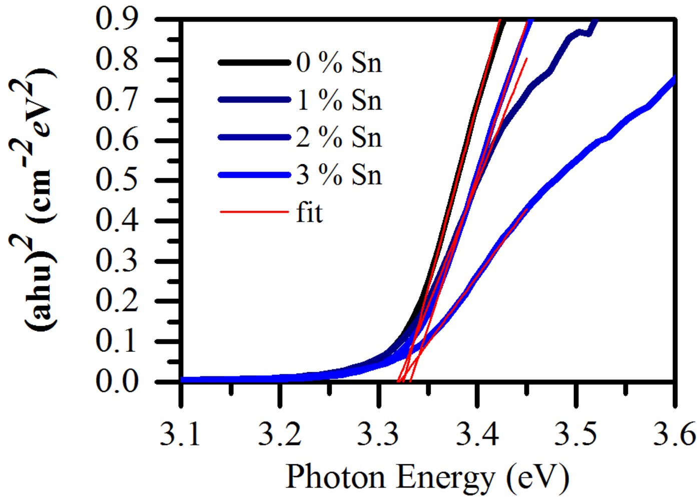

Figure S5| Tauc plot obtained from absorbance spectra of differently Sn doped ZnO NWs. Fits are presented as red lines.

Solar cell characteristics

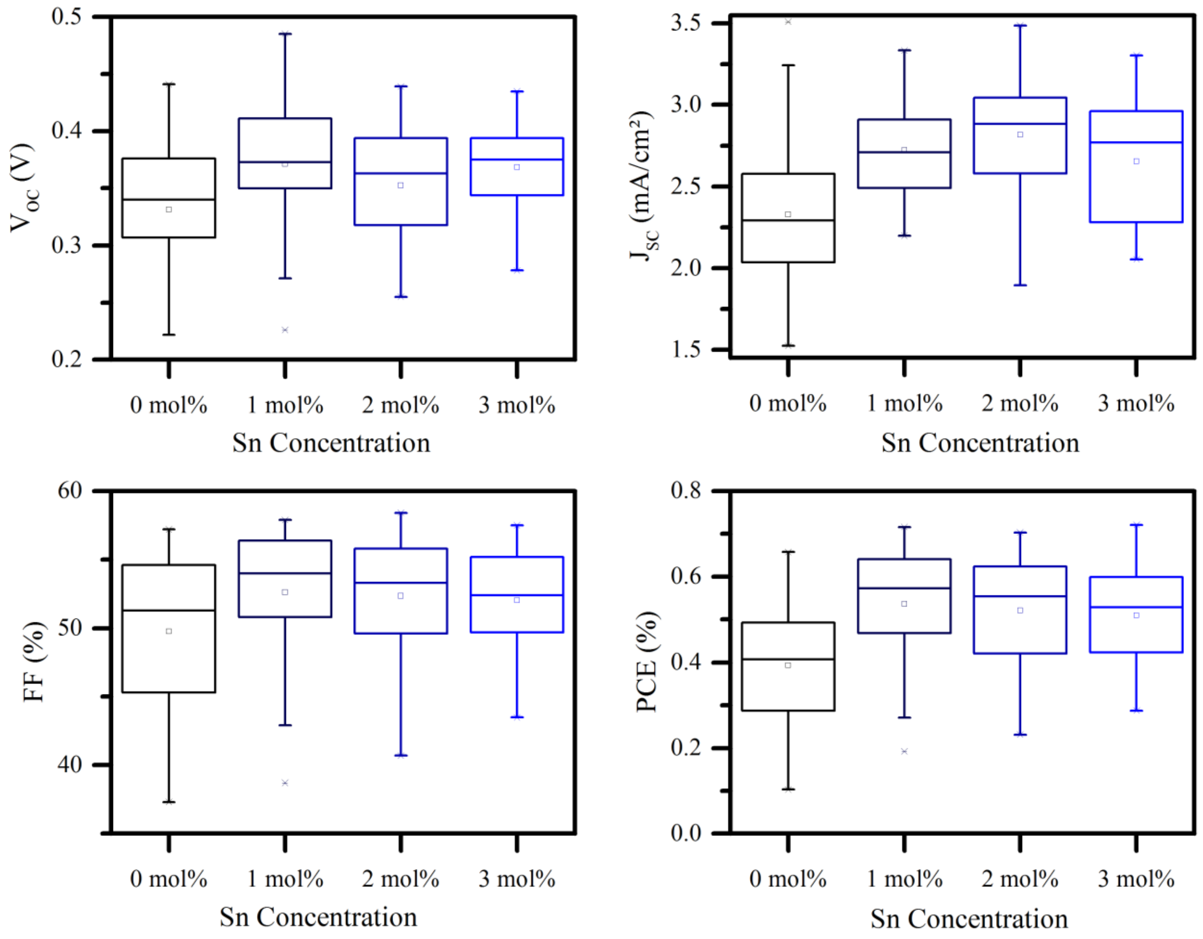

Figure S6 | Comparison of pure and doped HSC device performance (a) Voc, (b) Jsc, (c) FF, and (d) PCE. The average values presented here represent the statistical distribution of more than 40 solar cells of each kind out of 12 different and independent batches. Boxes indicate the interquartile range between the 75th and 25th percentile. Whiskers indicate the 95th percentile (upper) and 5th percentile (lower) while mean values are signed as squares and the median by the horizontal line.

Charge Collection Efficiency

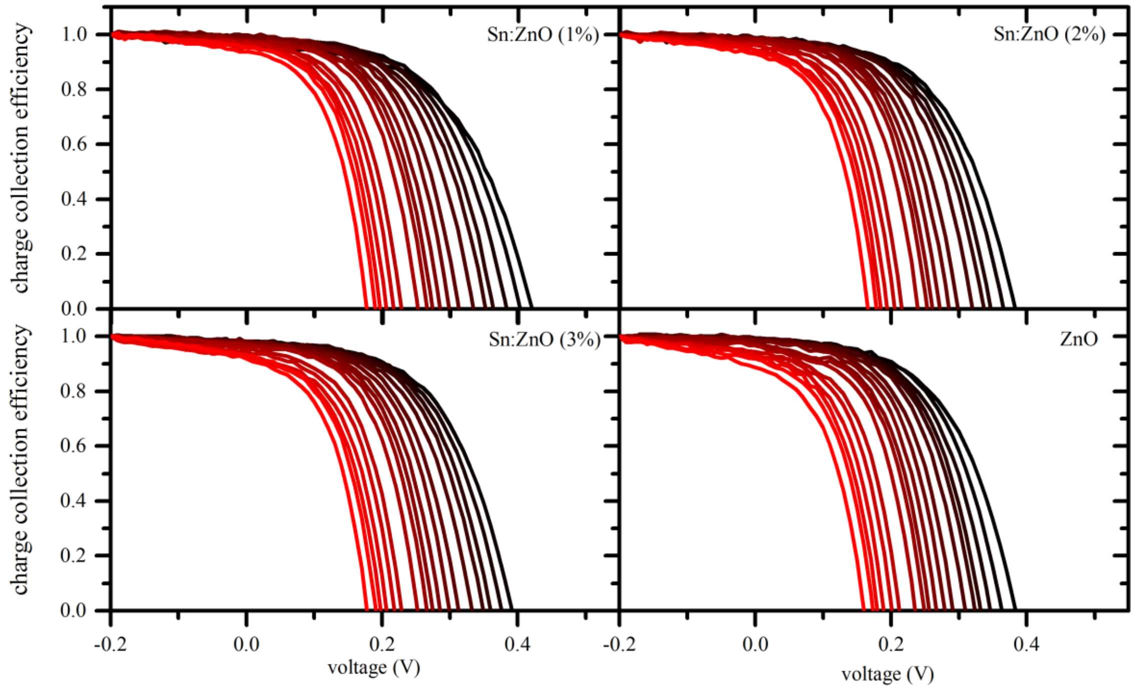

Figure S7| Charge collection probability calculated for J-V curves by J(V)/J(V=-0.2V). Measurements have been performed under different light intensities between 1-100 mW/cm² (from red to black color) for all devices under investigation. No matter how strong ZnO is doped, at J_{SC} charges can reach the electrodes efficiently and a voltage dependent photocurrent is basically absent.

Space-charge analysis

Light intensity dependent J-V measurements were carried out to investigate the device behaviour on varying charge carrier densities. This is particularly important for investigations on space charge effects. If the photocurrent density is linearly dependent on light intensity *I*, (and, thus, on the charge generation rate), there is no space charge region present in a working device and the photocurrent density can be expressed by:

$$J_{ph} = qGL = G(\mu_h\tau_h)^{1/2} V^{1/2} \qquad (1)$$

where J_{ph} is the photogenerated current density, q is the electric charge, G is the generation rate of electron-hole pairs, L is the specimen thickness, μ_h is the charge carrier mobility of holes, τ_h is the charge carrier lifetime, and V is the voltage across the entire photoactive layer. Note, G is direct proportional to I. The current density in the space charge limited conduction regime (J_{scl}) can be expressed by Mott-Gurney's law:

$$J_{scl} = \frac{9}{8}\varepsilon_r\varepsilon_0\,\mu_h \frac{V_1^2}{L_1^3} \qquad (2)$$

where ε_r and ε_0 represent the relative dielectric constant and the vacuum permittivity, respectively. Note that almost the entire voltage V drops on a region L_1 of hole accumulation therefore $V = V_1$ and the generated photocurrent in this region is essentially the total current. In the case of a space charge limited photocurrent, the width of L_1 can be determined from Equation 3 and 4:

$$qGL = \frac{9}{8}\,\varepsilon_r\varepsilon_0\,\mu_h\frac{V_1^2}{L_1^3} \tag{3}$$

$$L_1 = \left(\frac{9\varepsilon_r\varepsilon_0\mu_h}{8qG}\right)^{1/2} V_1^{1/2} \tag{4}$$

Consecutively, in case of accumulated charge carriers, i.e., the presence of a space charge region, the photocurrent density will show a three-quarter power law dependency on charge carrier generation $G^{3/4}$ (Equation 5), and simultaneously a square root dependency on voltage:

$$J_{ph} = qGL = q\left(\frac{9\varepsilon_r\varepsilon_0\mu_h}{8q}\right)^{1/4} G^{3/4}\,V^{1/2} \tag{5}$$

where V is the entire voltage drop on the region of hole accumulation, and L_1 is the length of the hole accumulation region. The photocurrent density (J_{ph}) of the pure and doped devices are plotted in Figure S8 as a function of effectively applied voltage (V_{eff}). The figure shows J-V curves for two different light intensities, namely 25 and 100 mWcm^{-2}. The effective voltage is approximated as $V_{eff} = V_{oc}$-V, where V_{oc} is the open circuit voltage at which $J_{ph} = 0$, V is the applied bias voltage, and $J_{ph} = J_L$, i.e. the current density under illumination. These curves basically consist of three regimes. If there is a large reverse bias applied and low forward bias across the solar cell, charges are sufficiently swept out without recombination leading to a saturation current. As forward bias increases, the effective built-in voltage decreases and charge carriers start to accumulate due to a mismatch of charge carrier mobilities. Here the current density shows a square root dependence on the effective voltage. At high forward voltages, the current is proportional to the applied voltage and therefore independent of the compensation voltage.

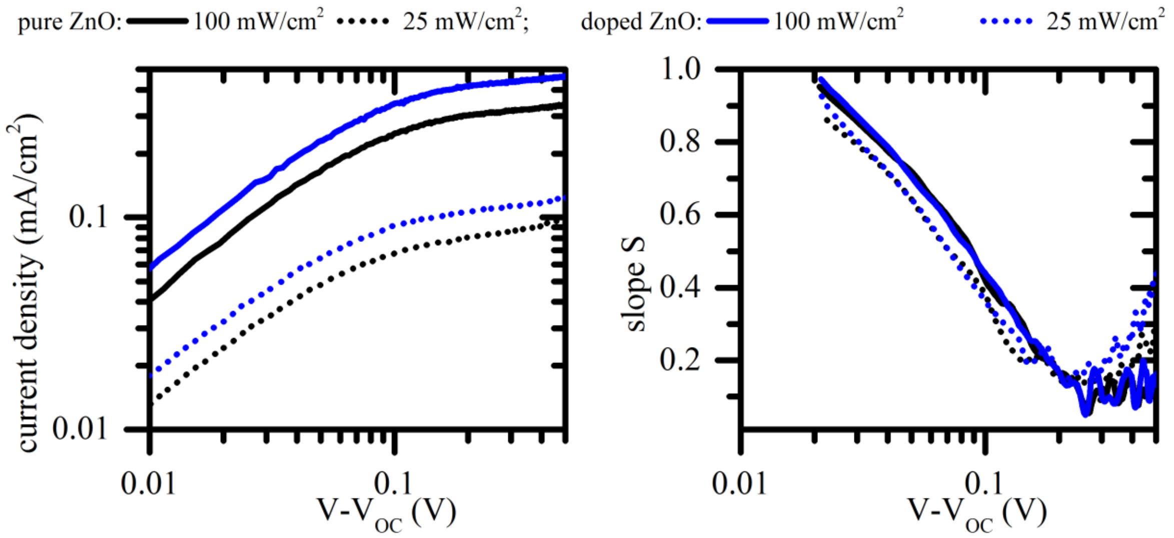

Figure S8| a) J_{ph}-V_{eff} characteristics of the doped and undoped ZnO NWs in hybrid solar cell under illumination at room temperature. b) S defined as the slope of the current density vs. effective voltage plot in order to elucidate its dependency and extract different transport regimes

As can be seen in Figure S8, doping does not result in a narrowing of the space charge region and also the transition to the saturation regime is identical between a non-doped and a strongly doped device. More importantly, however, this observation does not change if the incident light intensity is decreased by a factor of 4.

Although we could successfully decrease the electron mobility by Sn^{4+} doping and the NW conductivity, a reduction of space charge effects is virtually absent.
